# Identifying Skill and Usability Barriers to Digital Health Tool Use Among Older Adult Patients in US Safety Net Clinics: Mixed Methods Study

**DOI:** 10.2196/78430

**Published:** 2026-05-04

**Authors:** Taylor Rapson, Magaly Ramirez, Sandy He, Jeanette Wong, Hyunjin Cindy Kim, Isabel Luna, Andersen Yang, Junhong Li, Paul A Fishman, James D Ralston, Courtney R Lyles, Elaine C Khoong

**Affiliations:** 1 Department of Health Systems and Population Health, School of Public Health, University of Washington, Seattle, WA, 98195, United States; 2 Division of General Internal Medicine at Zuckerberg San Francisco General Hospital, Department of Medicine, University of California, San Francisco, 1001 Potrero Avenue, Main Building 5, 1st floor, 1M, San Francisco, CA, 94110, United States, 1 628-206-8000; 3 Action Research Center for Health, Zuckerberg San Francisco General Hospital, University of California, San Francisco, San Francisco, CA, United States; 4 Kaiser Permanente Washington Health Research Institute, Seattle, WA, United States; 5 Center for Healthcare Policy and Research, University of California at Davis, Sacramento, CA, United States; 6 Division of Clinical Informatics and Digital Transformation (DoC-IT), Department of Medicine, University of California, San Francisco, San Francisco, CA, United States

**Keywords:** digital skills, usability, digital equity, digital health technology, digital disparities, patient portal, eHealth, digital literacy

## Abstract

**Background:**

Despite their benefits, digital health tools often face adoption barriers because of the digital divide. Identifying the fundamental user skills required to effectively navigate these tools and the usability barriers is essential to addressing disparities in use.

**Objective:**

This study aimed to identify the skill and usability barriers to using digital health tools.

**Methods:**

This study included English-, Spanish-, or Cantonese-speaking patients, aged ≥50 years, who received care at an urban safety net health system in the United States. Participants completed a survey examining sociodemographic characteristics and digital health tool use and were observed and video recorded as they navigated four digital health care tasks: (1) launch a video visit, (2) visit a health website through a URL, (3) log in to the patient portal, and (4) sign up for a patient portal account. Participants who could not independently perform the tasks received additional support. Tasks were conducted in English, while instructions and additional assistance were provided in each participant’s preferred language. Video recordings were thematically coded to identify the fundamental skills needed for effective digital tool use and usability barriers in the design of digital tools. We examined whether task independence was associated with participant demographics and thematic categories using Kruskal-Wallis, *χ*^2^, and Fisher exact tests.

**Results:**

In total, 74% (34/46), 52% (31/60), 71% (44/62), and 70% (43/61) of participants (N=64) independently completed digital tasks 1, 2, 3, and 4, respectively. Older age, minoritized races and ethnicities, non-English language preference, lower educational attainment, access to cellular data only or no internet access, and lack of a portal account were associated with a higher likelihood of requiring assistance or being unsuccessful at completing each task (*P*<.001, except for older age [*P*=.004]). The qualitative coding of video recordings identified 3, 4, and 6 categories of typing, navigation, and human-computer interaction (HCI) skills, respectively, as fundamental skills required to independently complete digital tasks. *χ*^2^ and Fisher exact tests indicated significant associations between most typing, navigation, and HCI categories and independent task completion. We coded usability barriers as one of 6 learnability challenges or 3 operability challenges.

**Conclusions:**

This study identified that independent use of digital health tools requires fundamental typing, navigation, or HCI skills as well as high usability of digital tools. The inclusion of 4 different digital tasks added specificity to the type of skills and usability considerations necessary to ensure accessibility of digital health tools to diverse older adults. This study underscores the need for vendors to cocreate digital health tools with historically excluded end users in mind. As health care systems expand digital tool adoption, they must distinguish fundamental skill gaps from usability barriers, as each may require different intervention strategies.

## Introduction

Electronic health records, patient portals, telemedicine, and eHealth (often referred to as digital health tools) have increased the opportunities for individuals to engage more directly in their own care. Access to these tools enables appointment scheduling, medication refills, and communication with health care providers, reducing some of the accessibility burdens of face-to-face interactions [1]. These tools have been linked to improved chronic disease outcomes [2-7]. However, there are documented differences in their use among marginalized populations who may face individual and systemic barriers that hinder adoption [8-11]. Previous research has demonstrated different rates of digital health tool use among individuals and groups from minoritized racial and ethnic backgrounds (including those who prefer languages other than English), age groups, and genders, as well as differences in use for those who report barriers to broadband internet, devices, or data (eg, due to cost) [2,8-14]. In particular, older adults, especially those with non-English language preferences or who identify as a racial or ethnic minority, experience large disparities in digital health tool adoption because of steep disparities in device, internet, or cellular data access [9,14,15]. For example, the Pew Research Center reported that, according to a 2025 survey, although 97% of adults younger than 50 years owned a smartphone, only 78% of those 65 years and older owned a smartphone [16]. The Pew Research Center also found that while 81% of White adults in the United States had home broadband, only 71% of Black adults and 68% of Hispanic adults had it. Similarly, Black (19%) and Hispanic (28%) participants were more likely to rely on their smartphones for internet access (which may be less stable than broadband) than White (13%) or English-speaking Asian (11%) participants [16]. This digital divide may be exacerbated in health care settings that rely on digital health tools in routine practice, which is becoming increasingly common [10,13,15,17]. Studies within the past 5 years show that nearly 90% of US health care systems offered telehealth services, especially nonprofit hospitals, which have adopted telemedicine at a higher rate than other health systems [18,19].

To address these barriers for marginalized populations, there is a need for pragmatic research rooted in identifying and understanding digital literacy skills and user needs [20-23]. There is a growing body of literature documenting the self-reported digital literacy skills needed to access digital tools, yet the fundamental user skills required to effectively navigate these tools have not been well defined [23-27]. Self-reported digital literacy skills refer to a user’s perceived ability or confidence to perform general digital tasks and frequency of engagement, such as confidence in sending emails or frequency of using search engines. However, these metrics fail to delineate the fundamental knowledge and skills required to engage successfully with digital tools [25-27]. Fundamental knowledge and skills, in contrast, are the observable digital abilities required to navigate and interact with *any* digital device or system that must be present to successfully complete a digital task, such as being able to navigate a web browser, knowing how to open device apps, or recognizing clickable links [25-27]. Observational data are necessary to identify the fundamental knowledge and skills that impact self-reported digital literacy. To our knowledge, no studies have evaluated the fundamental digital knowledge and skills that individuals need to use digital health tools, particularly in marginalized communities disproportionately affected by the digital divide.

It is also important to identify usability barriers—which arise from poor product design—that prevent individuals from successfully interacting with digital tools and contribute to disparities in digital health tool uptake and use [17,22,28-30]. These usability challenges can impact both patients and clinicians. Clinical care team members may not be comfortable with workflows or tools for using eHealth or offering and supporting patients in using digital health tools [31]. Poorly designed interfaces, unclear navigation paths, and accessibility issues disproportionately impact individuals with limited digital literacy, disabilities, or language barriers [11,17,29,30,32]. These usability challenges, which often arise from limited patient participation in co-designing a digital health tool, can create unnecessary friction, discourage continued engagement, and deter patients from using digital health tools to engage in continuity of care [11,30,33]. Moreover, individuals from marginalized communities are less often represented in usability studies, limiting our understanding of the specific challenges they face when navigating these tools and hindering the development of equitable interventions to build the skills essential to improve the uptake of digital health tools [21,33]. Pragmatic human-centered research rooted in identifying the usability barriers for marginalized populations, using representative sampling within health care systems outside academic medical centers, may reveal key themes to improve user experience and mitigate disparities.

To address these gaps in the literature, we conducted a mixed methods study among multilingual older adults receiving care at an urban safety net health system. The aim of this study was to assess patient characteristics associated with successful completion of digital health tasks and to identify the skill and usability barriers that prevented successful completion. Using observational data of participants interacting with real-world digital health tools, we focused on the qualitative identification of skills and usability barriers mapped to existing frameworks. We then linked these qualitatively identified skills and barriers to the observed completion rates of digital health tasks (such as completing the process of logging in to a patient portal) to pinpoint the fundamental skills necessary to complete each digital health task independently and the usability barriers that many individuals face when navigating these tasks. We also examined the task completion rates overall and by key sociodemographic characteristics to assess disparities in task completion success.

## Methods

### Study Setting

This study was conducted within the San Francisco Health Network (SFHN), San Francisco County’s safety net health system encompassing 14 community-based primary care clinics. The SFHN serves a racially and ethnically diverse patient population of about 60,000, most of whom are enrolled in Medicaid or citywide insurance plans. The SFHN patient population is primarily non-White (80%); notably, 45% do not identify English as their primary language (35% prefer Spanish and >10% prefer Cantonese exclusively), and more than half report barriers to accessing and using digital health technology [34,35].

### Recruitment and Participation

Individuals were eligible for participation in this study if they met the following inclusion criteria: (1) receiving primary care at an SFHN clinic; (2) being aged 50 years or older; (3) speaking English, Spanish, or Cantonese as their primary language; (4) and not having a cognitive or behavioral impairment that would preclude them from providing informed consent or completing the digital tasks. We focused on adults aged 50 years or older, given the prevalence of digital barriers within this population. We focused on these 3 languages, as these are the most spoken languages among patients at SFHN, where our study was conducted. Between April 2023 and June 2024, patients at SFHN who met these inclusion criteria were recruited by the study team over the phone after receiving prior approval from their primary care providers. Study team members recruited 101 eligible patients who subsequently completed a participant questionnaire and performed the digital tasks.

### Ethical Considerations

The study was approved by the UCSF institutional review board (#22‐36493). A subsample of 64 participants provided written consent to record their progress through each digital task for this study. Participants were advised of their right to stop taking part in the study at any time. Participant questionnaires, observation notes, and video recordings were deidentified using study identification numbers during data collection. All physical documents with data were stored in a locked cabinet in a secure location. All electronic data were stored on Research Electronic Data Capture (REDCap; version 13.4.10; Vanderbilt University) and on servers accessible only to the study team. Participants were compensated US $20 for completing the questionnaire and US $40 for completing up to 4 digital tasks while being observed.

### Data Collection

The participant questionnaire, administered in the participant’s preferred language, included items eliciting information on participant age, sex, race, ethnicity, preferred language, comfort speaking English, and educational attainment and questions about device preference, access to a device with video capability, type of internet access, and current enrollment in the SFHN patient portal. Questionnaires were administered in person or over the phone by study staff. All digital task observations were conducted and video recorded in person. See Multimedia Appendix 1 for questions from the participant questionnaire included in this analysis.

After the participants completed the questionnaire, they were asked to use their preferred digital device, if on hand, or to choose a device provided by the research team that they felt comfortable using (either an Android smartphone, an Apple iPad, a Microsoft Windows laptop, or an Apple MacBook) to complete the digital task assessment. Participants were filmed as they attempted four digital tasks that simulated the necessary skills and actions required to use digital health technology effectively: (1) launching a video visit, (2) going to a specific health website, (3) signing in to a patient portal emulator using a dummy account, and (4) signing up for a patient portal account via the emulator. These tasks were selected due to their importance for engaging with digital health tools. While digital health tools have become more inclusive in recent years and offer the ability to switch between English and Spanish (and sometimes other languages) on the platform itself, many elements of these digital tools (such as automated text messages or notifications and prompts) are often unavailable in languages besides English; therefore, to replicate that experience, the platforms that the participants navigated to complete each task were in English only. Further details about the steps involved in each task are detailed in Multimedia Appendix 2, and specific instructions for each task are available online [40].

Participants were asked to independently complete each task by following a set of written instructions in their preferred language; if they were unsuccessful after 2 attempts, research staff provided a standardized instructional handout or video, followed by tailored, language-concordant verbal assistance if necessary. After each step of assistance, participants were asked to try the task again; if they were unsuccessful again, the research staff proceeded to the next level of assistance. Participants were deemed unsuccessful if they were unable to complete the task after receiving tailored one-on-one assistance. The level of assistance provided was logged by the research team member conducting the assessment. For accuracy, a second team member reviewed video recordings and assigned their score. If the 2 scores differed, discrepancies were addressed via team discussion.

The participant questionnaire, written task instructions, and assistance from the research staff were administered in the participant’s preferred language, as determined during recruitment. The questionnaire was either verbally administered by an interviewer proficient in the participant’s preferred language or completed as a paper questionnaire translated into their preferred language. Additionally, the interviewers provided translated instructions and, if needed, delivered verbal assistance.

### Analysis

#### Qualitative Analysis

For the qualitative analysis, we used framework analysis—a matrix-based method that allows for systematic comparison of themes across cases while retaining case context—to identify both the fundamental skills required of users and the usability barriers that prevented successful use [36]. First, we watched a subsample of video observations to gain a comprehensive understanding of the participants’ experiences and behaviors across the digital tasks. This initial stage helped to identify recurrent ideas, patterns, and areas of interest (typing skills, navigation skills, and human-computer interaction skills). We then developed a thematic framework to guide our analysis by generating a priori coding categories guided by the research questions (“what are the specific and foundational skills a user needs to successfully use digital health tools?” and “what are the usability barriers of these tools?”) and the existing literature about digital literacy and usability [27,32,36-38]. We categorized qualitative codes according to the level of digital literacy they reflected and distinguished them as novice or advanced, where appropriate. This categorization was informed by the digital literacy framework of van Deursen et al [27] and modified to appropriately reflect the skills pertinent to using digital health tools rather than the internet in general. This framework differentiates operational, formal, informational, and strategic digital skills, which capture the range of competencies needed to use digital tools effectively [27]. We primarily focused on operational and formal skills, such as device handling, typing, and navigating digital interfaces, which were most relevant to the tasks in our study. Novice-level skills included novice keyboard skills and novice device skills, which were observed as operational inability or skill gaps when performing a task. Advanced-level skills included advanced typing and advanced device skills—skills that encompassed strategic problem-solving abilities, such as troubleshooting errors and intuitive device understanding. We also mapped the predetermined coding categories of learnability (the capability of digital health tools to enable the user to learn their application) and operability (the capability of digital health tools to enable the user to operate and control them) to challenges associated with completing digital tasks due to usability issues [38]. We also used an inductive coding approach to allow codes to emerge from the data to limit a priori bias. Inductive and deductive coding ensured that both anticipated and novel insights were captured.

Next, we compiled and organized the codes in a structured matrix format, which enabled us to summarize and compare the nature and context of responses, while preserving the richness of the video observations. Each row represented an individual participant, and each column presented the themes associated with a specific task (Figure S1 in Multimedia Appendix 2). As a calibration step, 3 independent coders watched a sample of the videos and created a templated summary for each task in the matrix tool. This was used to assess the template and confirm that the domains were intuitive; identify any missing or incorrectly labeled domains; clarify any issues; and ensure consistency in content, style, and organization across coders. During this process, actions that supported or deviated from these predicted codes led to additional codes, iteratively expanding upon and updating the codebook to include relevant concepts (see Multimedia Appendix 3). After finalizing the codebook and template, to assess coding consistency, 3 members of the research team independently coded 25% of the recordings. After ensuring >85% agreement, the 3 members independently coded the remaining recordings.

#### Quantitative Analysis

For the quantitative component, survey responses were first exported from REDCap for preliminary descriptive summaries, focusing on the variables age, sex, race and ethnicity, preferred language, educational attainment, access to a device with video capability, source of internet access, and enrollment in the SFHN patient portal. These sociodemographic characteristics comprised the covariates for analysis.

Our primary quantitative outcome variable was task completion, which assessed whether a participant independently performed the task only with simple written instructions, performed the task after receiving standard assistance (ie, detailed handouts and video tutorials), performed the task after receiving tailored assistance (ie, verbal instructions and one-on-one help), or was unsuccessful. For analyses, we dichotomized this outcome, categorized as performing a task independently versus requiring any support (standard or tailored) or being unsuccessful (examples of these materials are available online) [39]. This cut point was chosen, as it represents the key distinctions between a user demonstrating fundamental skills and those requiring further assistance or training. Because of technical issues (eg, malfunctioning cameras or recording failures) and participants occasionally declining tasks that they found too difficult, the number of video recordings varied slightly across tasks, as individuals without recordings were treated as missing data and removed from the analysis for that task.

We conducted global tests of association to evaluate relationships between participant sociodemographic characteristics and task completion across all 4 tasks. Kruskal-Wallis tests were used to test the association between age—modeled as a continuous variable—and task completion. *χ*^2^ tests of independence were used for categorical sociodemographic variables, and Fisher exact tests were used when the expected cell counts were <5. These tests evaluated whether task completion status differed by sociodemographic characteristic when considering all tasks jointly, rather than within a single task. R software (version 4.3.3; R Foundation for Statistical Computing) was used to conduct all analyses.

#### Comparison of Qualitative Findings and Quantitative Data

To assess the relationship between identified themes and task completion, qualitative and quantitative analyses were conducted in parallel using a convergent triangulation design in which the datasets were integrated prior to the combined analysis [40-42]. Key qualitative themes were grouped into higher-order categories to facilitate interpretation. This involved identifying conceptual connections between codes and organizing them into thematic categories. These thematic categories were then converted into categorical variables and incorporated into the quantitative dataset. Triangulating the 2 types of data supported the identification of foundational skills necessary to use digital health tools successfully (and, conversely, the skill gaps that required interventional support). We used *χ*^2^ tests of independence and Fisher exact tests to determine the significance of associations between each identified skill or skill gap and the task completion outcome. Fisher exact tests were used when the expected cell counts were <5.

## Results

### Participant Characteristics

In total, 64 participants, comprising 40 men and 24 women, provided consent for the video recording of their digital task completion efforts. The language spoken by most participants (n=42, 65.6%) was English, followed by Cantonese (n=14, 21.9%) and Spanish (n=8, 12.5%). The mean age was 61.9 (SD 7.4; range 51.0‐83.4) years. Notably, 79.7% (51/64) of the participants identified as non-White, and 53.1% (34/64) of the participants had an educational attainment of a high school degree or less. Most participants (55/64, 85.9%) strongly preferred using their smartphone, with some preferring a desktop or laptop computer (12/64, 18.8%) or tablet (9/64, 14.1%), and only 1.6% (1/64) preferred using a simple cell phone. Moreover, 90.6% (58/64) of the participants owned a device with video capability. Of the 64 participants, 60.9% (n=39) had both Wi-Fi and unlimited data on their smartphones, whereas 23.4% (n=15) used Wi-Fi only and 10.9% (n=7) had unlimited data only. Lastly, 65.6% (42/64) of the participants were enrolled in the patient portal at SFHN. Participant characteristics are displayed in Table 1.

**Table 1.** Participant descriptives.

Characteristic	Subsample with video observations (N=64)
Age (years), mean (SD)	61.9 (7.4)
Male sex (%)	62.5
Race and ethnicity, n (%)	
Non-Hispanic White	12 (18.8)
Non-Hispanic Black or African American	10 (15.6)
Non-Hispanic Asian	21 (32.8)
Non-Hispanic other or multiple races	4 (6.3)
Hispanic or Latinx	16 (25.0)
Decline to answer	1 (1.6)
Preferred language, n (%)	
English	42 (65.6)
Spanish	8 (12.5)
Cantonese	14 (21.9)
Education, n (%)	
High school (no degree) or less	18 (28.1)
High school degree or GED^a^	16 (25.0)
Some college or associate’s degree	18 (28.1)
Bachelor’s degree or higher	12 (18.8)
Own device with video capability, n (%)	
Yes	58 (90.6)
No/don’t know	6 (9.4)
Preferred device(s)^b^, n (%)	
Smartphone	55 (85.9)
Desktop or laptop computer	12 (18.8)
Tablet	9 (14.1)
Simple cell phone	1 (1.6)
Internet access, n (%)	
Both Wi-Fi and unlimited data on phone	39 (60.9)
Wi-Fi only	15 (23.4)
Unlimited data on phone only	7 (10.9)
Neither/don’t know	3 (4.7)
Enrolled in patient portal, n (%)	
Yes, at SFHN^c^	42 (65.6)
No/don’t know	22 (34.4)

a GED: General Educational Development.

b The categories are not mutually exclusive.

c SFHN: San Francisco Health Network.

### Task Completion

Task completion rates per task are displayed in Table 2. As shown in Table 2, 46 to 62 participants were filmed while completing the 4 digital tasks. Independence in task completion was the highest for task 1 (34/46, 74%) and lowest for task 2 (31/60, 52%).

Global tests of independence were used to evaluate associations between participant characteristics and task completion across all 4 tasks. Age, race and ethnicity, preferred language, educational attainment, access to a device with video capability, source of internet access, and enrollment in the patient portal were significantly associated with task completion. Participants who were older were more likely to require assistance or be unsuccessful across tasks (*P*=.004). Participants who were White were significantly more likely to complete each task independently than participants of other races and ethnicities (*P*<.001). Similarly, preferred language was associated with task completion, with those who preferred Spanish or Cantonese more likely to require assistance or be unsuccessful than those who preferred English (*P*<.001). Participants with a high school education or less were also more likely to struggle to complete the tasks independently and had higher rates of being unsuccessful or requiring assistance (*P*<.001). Access was also significantly associated with task completion, with those who had access to a device with video capability and Wi-Fi more likely to complete tasks independently than those without video capability (*P*=.05) or who relied on data to access the internet (*P*<.001). Individuals already enrolled in the patient portal tended to be more successful and had a higher likelihood of completing each task independently than those who were not enrolled in the patient portal or were unsure (*P*<.001).

**Table 2.** Task completion rates for each task by key sociodemographic characteristics.

	Task 1 (n=46)		Task 2 (n=60)		Task 3 (n=62)		Task 4 (n=61)		*P* value^c^
	Indep^a^	A/U^b^	Indep	A/U	Indep	A/U	Indep	A/U	
Participants, n (%)	34 (74)	12 (26)	31 (52)	29 (48)	44 (71)	18 (29)	43 (70)	18 (30)	
Age (years), mean (SD)	60.2 (6.49)	67.2 (7.98)	60.8 (7.68)	62.7 (5.99)	61.1 (7.11)	62.3 (5.75)	60.5 (7.21)	63.5 (5.23)	.004^d^
Male sex (%)	58.82	66.67	64.52	62.07	68.18	44.44	69.77	38.89	.06
Race and ethnicity, n (%)									.001
NH^e^ White	8 (23.5)	1 (8.3)	8 (25.8)	4 (13.8)	12 (27.3)	0 (0)	12 (27.9)	0 (0)	
NH Black or African American	4 (11.8)	3 (25.0)	6 (19.4)	4 (13.8)	9 (20.5)	2 (11.1)	9 (20.9)	2 (11.1)	
NH Asian	10 (29.4)	5 (41.7)	8 (25.8)	10 (34.5)	11 (25.0)	8 (44.4)	11 (25.6)	7 (38.9)	
NH other or multiple races	3 (8.8)	0 (0)	3 (9.7)	1 (3.4)	4 (9.1)	0 (0)	4 (9.3)	0 (0)	
Hispanic or Latinx	9 (26.5)	3 (25.0)	6 (19.4)	10 (34.5)	8 (18.2)	8 (44.4)	7 (16.3)	9 (50.0)	
Preferred language, n (%)									.001
English	23 (67.6)	5 (41.7)	26 (83.9)	13 (43.3)	37 (84.1)	5 (27.8)	36 (83.7)	6 (33.3)	
Spanish	4 (11.8)	2 (16.7)	0 (0)	8 (26.7)	3 (6.8)	5 (27.8)	2 (4.7)	6 (33.3)	
Cantonese	7 (20.6)	5 (41.7)	5 (16.1)	8 (30.0)	4 (9.1)	8 (44.4)	5 (11.6)	6 (33.3)	
Education, n (%)									.001
High school degree, GED^f^, or less	16 (47.1)	11 (91.7)	10 (32.3)	22 (75.9)	29 (65.9)	17 (94.4)	16 (37.2)	15 (83.3)	
Some college or higher	18 (52.9)	1 (8.3)	21 (67.7)	7 (24.1)	15 (34.1)	1 (5.6)	27 (62.8)	3 (16.7)	
Own device with video capability, n (%)									.05
Yes	34 (100)	9 (75.0)	30 (96.8)	26 (89.7)	40 (90.9)	16 (88.9)	40 (93.0)	15 (83.3)	
No/don’t know	0 (0)	3 (25.0)	1 (3.2)	3 (10.3)	4 (9.1)	2 (11.1)	3 (7.0)	3 (16.7)	
Internet access, n (%)									.001^g^
Both Wi-Fi and unlimited data	23 (67.6)	5 (41.7)	19 (61.3)	18 (62.1)	28 (63.6)	10 (55.6)	28 (65.1)	9 (50.0)	
Wi-Fi only	8 (23.5)	3 (25.0)	11 (35.5)	2 (6.9)	11 (25.0)	3 (16.7)	10 (23.3)	4 (22.2)	
Unlimited data on phone only	3 (8.8)	1 (8.3)	1 (3.2)	6 (20.7)	5 (11.4)	2 (11.1)	5 (11.6)	2 (11.1)	
Neither/don’t know	0 (0)	3 (25.0)	0 (0)	3 (10.3)	0 (0)	3 (16.7)	0 (0)	3 (16.7)	
Enrolled in patient portal, n (%)									.001
Yes, at SFHN^h^	28 (82.4)	3 (25.0)	27 (87.1)	13 (44.8)	33 (75.0)	8 (44.4)	34 (79.1)	7 (38.9)	
No/don’t know	6 (17.6)	9 (75.0)	4 (12.9)	16 (55.2)	11 (25.0)	10 (55.6)	9 (20.9)	11 (61.1)	

a Indep: independent completion of task.

b A/U: assisted or unsuccessful completion of task.

c *χ*2 test was used to assess differences by sociodemographic characteristic, unless otherwise specified; *P≤*.05 was considered significant.

d Kruskal-Wallis test was used to assess differences by age; *P*≤.05 was considered significant.

e NH: non-Hispanic.

f GED: General Educational Development.

g Fisher exact test was used to assess differences by internet access; *P*≤.05 was considered significant.

h SFHN: San Francisco Health Network.

### Video Observations

#### Thematic Coding

**Figure 1.**
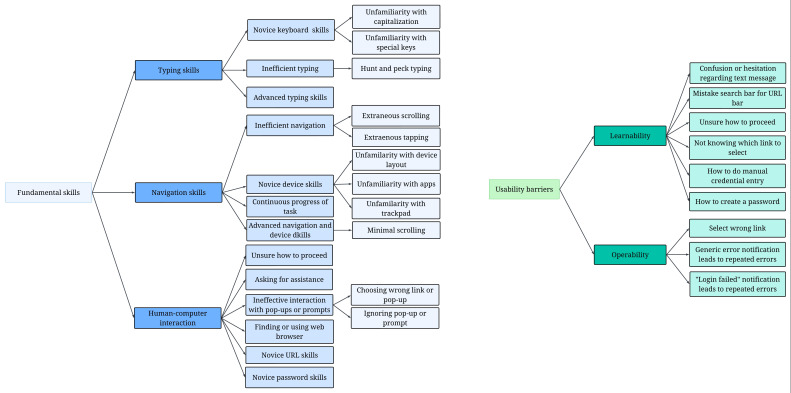
Qualitative thematic maps for fundamental skills and usability barriers.

The following findings were based on 91% agreement between the 3 coders for the first 15 participants and independent coding of the remaining videos. Thematic findings were grouped into factors, mapped to the overall dimensions of fundamental skills and usability barriers, and reorganized for interpretation. To ease analysis, we decided to separate the codes representing usability barriers from those representing fundamental skills, with several codes representing skills and usability barriers. Figure 1 shows an overview of themes generated during the observational coding process, with subcategories within each key theme, and separated by fundamental skills and usability barriers.

#### Fundamental Skills

Video observations revealed that participants exhibited a range of digital skills from novice to advanced. The coding matrix categorized participant behaviors into key themes, including typing skills, navigation skills, and human-computer interaction. Typing skills referred to a participant’s ability to navigate the keyboard (particularly capitalizing letters and finding special keys) and demonstration of inefficient typing (“hunting” and searching for letters) or advanced typing skills (proficiency with typing, capitalization, and special keys). Navigation skills comprised inefficient navigation (eg, extraneous scrolling or tapping on the device), novice device skills (eg, unfamiliarity with device layout or apps), advanced navigation and device skills (often indicated by minimal scrolling), and continuous progress of the task. Human-computer interaction (HCI) encompassed codes that demonstrated the way participants engaged or did not engage with their devices to complete the tasks, ranging from how they responded to prompts; whether they were unsure how to proceed, requested assistance, or ineffectively interacted with pop-ups or prompts; to having trouble with specific elements of the tasks (eg, novice URL skills, trouble finding or using a web browser, or novice password skills).

A significant proportion of participants exhibited novice-level digital skills, particularly in typing and navigation. Many participants relied on inefficient typing techniques, such as hunt-and-peck typing and relying on using one finger or a stylus to locate and enter subsequent characters. The inability to appropriately capitalize letters or locate and select special characters created significant barriers to completing the tasks independently. Similarly, many participants demonstrated navigation inefficiencies. Participants exhibited trouble navigating their device with extraneous and repeated tapping or scrolling, confusion locating specific apps or phone settings, or difficulty using a touchscreen or trackpad. In contrast, participants who demonstrated success at navigating the tasks independently almost exclusively displayed advanced navigation skills and the ability to progress through the tasks continuously without breaks in performance flow.

Many individuals also struggled with more complex HCI actions beyond simply navigating their device or using the keyboard. Common observations included participants not understanding how to proceed through the task because they struggled with fundamental concepts such as recognizing common digital icons (eg, settings icon, “close window” button, home icon, or back/forward arrows) or clicking the “next” button, having trouble finding or navigating to a web browser (or conceptually understanding what it is), ineffectively interacting with task pop-ups or prompts (eg, ignoring a pop-up/prompt or choosing the wrong link), and difficulty using the web browser (eg, not knowing how to select the web browser icon to open a new window or how to proceed once the web browser was open). Individuals also demonstrated novice URL and password skills, as they struggled with accurate text entry to successfully type in a URL for a website or enter the provided password. Difficulty performing complex tasks despite receiving instructions was a common reason why participants asked for assistance, often demonstrated even before they attempted the task or after several unsuccessful attempts to complete an action.

Table 3 presents the association between typing, navigation, and HCI codes and task performance. Most notably, across tasks, keyboard skills, navigation skills, and HCI skills showed a significant association between the factor and the ability to complete each task independently. Individuals who demonstrated unfamiliarity with navigating the keyboard, particularly with how to capitalize letters or find special characters, always required assistance or were unsuccessful at completing the digital tasks (*P*<.001 in all cases). Inefficient navigation with extraneous scrolling or tapping of their device was strongly associated with needing assistance or unsuccessfully completing the task, particularly in task 1 (*P*=.001), task 2 (*P*<.001), and task 4 (*P*=.006), which required more complex device navigation to complete the task. Novice device skills, demonstrated by lack of familiarity with one’s device, whether it be the layout, finding and using apps, or navigating the trackpad, were significantly associated with requiring assistance or unsuccessfully completing the task across tasks (task 1, *P*<.001; task 2, *P*<.001; task 3, *P*=.004; task 4, *P*=.002). Typing skills (both inefficient typing and advanced typing skills) were not significantly associated with digital task completion (exact *P* values given in Table 3). However, we did not assess speed to completion.

The complexity of the digital tasks required several fundamental human-computer interaction skills, beyond navigating one’s device, to complete each task. Displaying confusion or hesitation with how to proceed during a task was a significant barrier for individuals during each task, ranging from not being sure how to proceed to confusion about how to navigate barriers that arise or where to put information (tasks 1, 2, and 4, *P*<.001; task 3, *P*=.02). Individuals also often asked for assistance when stuck on a task, demonstrating the complexity of progressing through a task as a barrier to independently completing a task (*P*<.001 in all cases). In task 2, the ability to find and use a web browser appropriately and the ability to type in a URL correctly were also significantly associated with being unsuccessful or requiring assistance to complete the task (*P*<.001 for both). In tasks 3 and 4, having trouble with creating a username or password (eg, not sure how to create one) was associated with needing assistance or unsuccessfully completing the task (task 3, *P*=.004; task 4, *P*=.006).

**Table 3.** Association between thematic fundamental skills codes and task completion.

	Task number	Independent completion^a^, n	Assisted/unsuccessful completion^a^, n	*P* value
Typing skills				
Inefficient typing (tasks 2‐4)	2	24	24	>.99
	3	25	17	<.001^b^
	4	28	15	.26
Novice keyboard skills (tasks 2‐4)	2	0	10	<.001^b^
	3	0	5	<.001^b^
	4	0	7	<.001^b^
Advanced typing skills (tasks 2‐4)	2	5	0	.05^b^
	3	7	0	.17^b^
	4	8	0	.09^b^
Navigation skills				
Inefficient navigation	1	4	8	.001^b^
	2	4	23	<.001
	3	11	9	<.001^b^
	4	11	12	.006
Novice device skills	1	2	10	<.001^b^
	2	2	21	<.001
	3	2	6	.004^b^
	4	2	7	.002^b^
Advanced navigation and device skills	1	28	1	<.001^b^
	2	29	1	<.001
	3	35	4	<.001^b^
	4	33	2	<.001^b^
Continuous progress of task	1	22	0	<.001
	2	24	0	<.001
	3	31	0	<.001
	4	23	0	<.001
Human-computer interaction				
Unsure how to proceed	1	7	12	<.001
	2	0	15	<.001
	3	4	6	.02^b^
	4	5	11	<.001^b^
Asking for assistance	1	3	12	<.001^b^
	2	1	19	<.001
	3	7	13	<.001
	4	8	13	<.001
Ineffective interaction with pop-ups or prompts	1	3	15	.006
	2	4	11	.04^b^
	3	8	11	.002
	4	12	13	<.001^b^
Finding or using web browser (task 2 only)	2	0	13	<.001
Novice URL skills (task 2 only)	2	3	14	<.001
Novice password skills (tasks 3 and 4 only)	3	5	8	.004^b^
	4	13	13	.006

a The independent completion and assisted/unassisted completion columns show the number of participants displaying each qualitative code per task.

b Indicates Fisher exact test; *χ*2 tests of independence used in all other instances. *P≤*.05 was considered significant.

#### Usability Barriers

Video observations also revealed that task completion was hindered by several usability barriers. The key usability barriers across all the tasks were related to learnability and operability. Tables4  5 summarize the learnability and operability issues for each task. In task 1, joining a video visit from a text message and unfamiliarity with system-generated notifications led to hesitation or errors in navigating the log-in prompt (characterized by the codes “confusion/hesitation regarding text messages” and “unsure how to proceed”), illustrating learnability barriers to joining video visits, despite the instructions in the text. In addition, individuals often did not select the video link, instead opting to choose an alternative method, such as replying to the text (despite it being a no-reply message) or attempting to join the video visit unsuccessfully. Additionally, once the participant successfully selected the link in the text message, additional pop-ups and links necessary to join the video call confused the participant, resulting in those pop-ups and links being ignored or ineffectively addressed. The operability limitation of this task was that the system did not provide clear enough cues for individuals to correctly select the link to join the video visit; therefore, individuals often did not know which link to select and instead selected the wrong link, either delaying their ability to join a video visit or preventing it altogether.

**Table 4.** Examples of learnability usability barriers by task.

Task	Thematic code	Example	Illustrative quote
1: sign in to a video visit	Confusion or hesitation regarding text messages Unsure how to proceed Not knowing which link to select	Clicking on a video call link from a text message requires a level of understanding (to click the link) that users might not have Results in hesitating to click the link, ignoring the text, or replying to a no-reply text Individual ignores necessary prompts for joining a video call or selects other incorrect links	“Ok so I have to copy that link and put it on my phone?” “So, I open it, I guess?” “Ok but what does…main menu? What does that look like?”
2: go to a specific website	Mistake search bar for URL bar How to do manual credential entry Unsure how to proceed Not knowing which link to select	Individual types the URL into the search bar instead of the URL bar Individual is not sure how to proceed once they type the URL in Individual does not select the appropriate link or button; instead, they select elsewhere	“I don’t know how to get to a website.” “If I put it up here, it doesn’t come down here.” [in reference to search bar] “Now I’m stuck.”
3: sign in to the patient portal	How to do manual credential entry Unsure how to proceed	Individual does not display the basic level of knowledge for password or username duplication (copying the given password/username into account) Individual stops after typing in password, without clicking enter to continue	“Oh, it says Patient, I’m the patient!” [in response to the username being “Patient123” after entering only “123”]
4: sign up for a patient portal account	How to create a password Unsure how to proceed	Individual does not display the basic level of knowledge necessary to create a password Individual does not know how to proceed once they type in a password or username	“Did it accept? Did it accept, or not?” “I don’t know what you mean by that.” [in response to being instructed to capitalize a letter] “Is that the password? Nothing? I have to create? Invent something?”

**Table 5.** Examples of operability usability barriers by task.

Task	Thematic code	Example	Illustrative quote
1: sign in to a video visit	Selecting wrong link	Individual clicks on a wrong link to join the video call	—^a^
2: go to a specific website	Generic error notification leads to repeated errors	Complexity and length of the URL results in errors difficult to discern and correct	[Participant mistakenly typed a forward slash instead of a backslash]
3: sign in to the patient portal	Sign-in process difficult to remember or master Generic error notification leads to repeated errors	“Login failed” notification does not indicate the error, resulting in difficulty correcting username or password errors	"Even if I signed in now, after I walk out the door I won't know how to do it again." "Those are the little things that make a difference." [when noticing that "s" in the password “AppleSauce” needs capitlizing]
4: sign up for a patient portal account	Activation code disrupts sign-up process Generic error notification leads to repeated errors	Activation code causes confusion and leads to error making “Login failed” notification does not indicate the error, resulting in difficulty correcting username or password errors	“I don’t know [where the activation code is].” “Where do I put [the activation code], right here?” "I'm confused.” [when unable to quickly identify error]

a Not applicable.

Task 2 was the most difficult for participants, with learnability and operability barriers affecting the ability to complete the task of signing in to the patient portal on a web interface. Many participants displayed misinterpretation of browser suggestions and confusion regarding where to type in the URL (coded as “mistake search bar for URL bar”) and the extent to which the URL needs to be complete (categorized as “how to do manual credential entry;” many stopped before typing in the complete URL or mistakenly used autofill to create a false URL), illustrating the learnability barriers associated with this task. In addition, individuals were often unsure how to proceed, sometimes selecting irrelevant links or failing to recognize the correct link to continue to the patient portal website. Typing in a URL with multiple characters and capitalizations also proved to be a user burden, as many individuals made small mistakes that led to repeated failures, without indications of where the error was, illustrating the operability barrier in URL design. These mistakes included typos in the URL or using autofill to automatically complete the URL, despite the autofill not matching the provided URL.

Participants struggled with the password aspects of tasks 3 and 4, further exposing learnability and operability barriers. They often misunderstood the login prompts, illustrating conceptual clarity issues in password duplication or creation for digital tools (categorized as “how to do manual credential entry” and “how to create a password”). The knowledge required to understand and replicate the complexity required for password creation is too steep, resulting in learnability barriers for many users who do not possess the basic level of knowledge necessary to navigate username and password duplication or creation. Additionally, in task 4, once individuals created a password, they often did not know what to do next to proceed, illustrating the learnability issues of progressing successfully through the steps required to sign in or sign up for a patient portal account. The operability limitation of these tasks was notifications that did not adequately notify the participant of their error, leading to repeated errors (categorized as “generic error notification leads to repeated errors” and “‘login failed’ notification leads to repeated errors”). For example, the activation codes were confusing and led to errors among participants without proper indication. When prompted that an activation code was needed, many did not know what it was, where to find it, or what to do with it. If they mistyped the activation code but not the username or password, they would receive only a “login failed” notification that did not properly indicate where the error occurred.

## Discussion

### Principal Results

Our study identified the fundamental skills necessary to be able to effectively navigate digital health tasks, found significant disparities in those skills among participants, and established usability issues associated with navigating digital health tools.

Although nearly universal access to digital devices has resulted in increased device ownership and personal use, only 53.1% (34/64) of participants were able to complete more than half of the tasks independently. Notably, 15.6% (10/64) were unable to complete any tasks independently, indicating a discrepancy between device ownership and ability to navigate these devices effectively, particularly for digital tasks related to health care. These data are supported by prior work assessing disparities in digital health tool use [9,11,43].

We expected that typing skills, navigation skills, and HCI skills would be associated with the ability to complete the digital tasks independently, but it was found that only a subset of these factors were associated with task completion, which may be limited by our sample size.

#### Challenges in Task Completion

Nearly half of the participants were able to complete each task independently, with 56.3% (36/64) able to complete tasks 1, 3, and 4 independently. The task of typing in a URL in task 2 posed unique barriers and resulted in more participants requiring assistance or being unsuccessful. This is potentially explained by the fact that typing in a complete URL is increasingly uncommon in digital health contexts, where users typically rely on search engines (eg, searching “SFHN MyChart patient portal” in the search bar), use autocomplete based on browsing history, or access the portal through an app. This is further supported by the fact that many participants struggled to recognize the URL bar or conceptually understand what was being asked of them during task 2. Despite this, many informational handouts about the patient portal continue to direct users to type in a specific URL to access the patient portal. These materials should consider providing alternative instructions that align with common user behavior, such as using search terms or app-based access.

#### Disparities in Task Completion

We anticipated that there would be disparities in task completion based on age, sex, race and ethnicity, preferred language, education, access to a device with video capability, source of internet access, and patient portal enrollment. Older age, minoritized races and ethnicities, preferred languages other than English, low educational attainment, possession of a device without video capability, data as the source of internet access, and not being enrolled in the patient portal contributed significantly to gaps in digital skills. These sociodemographic characteristics were significantly associated with requiring assistance or being unsuccessful, particularly for tasks that involved multiple steps or complex engagement with digital interfaces. This suggests that individuals from these groups may be disproportionately less likely to have the skills required to feel confident and able to complete each task independently. Moreover, tasks 3 and 4 were more complex, involving reading comprehension and following multiple prompts to enter pertinent text or create a unique password. These elements (such as log-in prompts and password creation details) were in English only, which posed obvious barriers to individuals who preferred non-English languages.

Although this study is not sufficiently powered to examine the intersectional vulnerabilities of participants who experience multiple, overlapping socioeconomic disadvantages—factors that may confound their ability to complete digital tasks independently—there remains a critical need for future research to adopt an inclusive approach to participant recruitment and analysis in usability studies. Specifically, examining how compounded forms of marginalization (eg, low income, limited education, language barriers, and age-related factors) shape digital engagement is essential for uncovering the full extent of challenges faced by individuals most at risk of digital exclusion. Such efforts are key to informing the design of equitable digital health tools and implementation strategies that promote broader and more effective adoption across diverse populations [17,21,22,33,44].

#### Challenges in Digital Proficiency

These results illustrate that the basic foundational skills necessary for successful navigation and use of digital health tools include competency with one’s device, such as knowing basic keyboard navigation and how to find appropriate apps or change settings, being able to navigate complex processes such as interacting with pop-ups and successfully navigating web browsers, and being able to proceed after each step. Without these basic foundational skills, an individual’s ability to successfully use and navigate digital health tools decreases significantly. Strategies for improving digital skills to ensure that individuals have the necessary foundational skills include implementing digital competency screening tools and providing digital skills workshops and trainings that emphasize hands-on practice with common tasks such as manual text entry, device and keyboard navigation, managing settings, and interacting with complex pop-ups or prompts. Prior work from our team has demonstrated that the brief, 9-item Skills Measurement and Readiness Training for Digital Health (SMART Digital Health) Scale is reliable for measuring skills for digital health-related tasks and activities and predicting the level of support an individual may need [23].

In addition, prior studies have found that providing support such as technical support or digital education alleviates some of the digital proficiency challenges faced by many and promotes the use of digital health tools, particularly among those who face greater barriers to digital use [45-47]. In addition to providing detailed assistance and training to address the complex barriers faced by those with limited digital literacy, ongoing or repeated support may be necessary, as one-time training or technical help may not be sufficient to overcome persistent technical challenges [47].

#### Usability Barriers May Impact Digital Task Success

The findings highlight the critical role of learnability and operability challenges across a range of digital health tasks commonly required for digital patient engagement. Participants encountered distinct barriers during each task, verbalizing frustration and making repeated assistance requests. Many of the difficulties were often tied to misunderstandings about how to initiate or complete a task, suggesting that digital inclusion efforts should simplify interfaces, provide clear prompts, and reduce extraneous steps to mitigate the usability issues faced by many participants.

Learnability issues can lead to a range of negative user experiences, including hesitation and confusion, a greater need for assistance and additional support to complete each task, and low user confidence. Each task required a level of understanding that users might not have to complete the task, resulting in a system that does not equally support users of all backgrounds to adapt and use the system. In addition, there were operability barriers across each task. Particularly, difficulties such as clicking the wrong link, making errors without descriptive or specific notifications, or being confused about how to proceed during a task result in a system that does not provide users with the necessary functionalities to allow users of all backgrounds to adapt and use the system. Efforts to improve the clarity of digital task prompts and processes, ranging from simplifying the URL to log in to the patient portal to clarifying how to successfully join a video visit from a text (or link) or how to engage with certain prompts, will enhance system learnability and operability and allow users to better engage with the system and remember how to use the system going forward.

These findings are consistent with prior research identifying digital navigation and account access as common friction points for users with limited digital literacy [9,11]. However, our human-centered design (HCD) approach to this study adds nuance by documenting how these challenges manifest in real time and vary by task complexity. Previous literature often examines digital competence in aggregate, but our task-specific analysis reveals that even users with some experience may struggle with unfamiliar or multistep processes, particularly when there are long task completion times or when confusing interfaces or prompts lead to repeated errors [48,49]. As adoption of digital health tools continues to increase, there are opportunities to use HCD approaches to identify usability barriers and align digital health tool design with user needs to ensure a more equitable distribution of digital health tool benefits [32]. This is particularly important, as there are numerous examples in which digital tools exacerbate inequities. HCD approaches can help uncover previously unknown health equity issues and drive efforts to address those inequities more acutely experienced by marginalized populations that are often not considered priorities in research [28-30,32,33].

#### Vendors Need to Prioritize Co-Design to Address Usability Barriers

The findings from this study underscore the pressing need for digital health vendors to take a more active role in addressing usability barriers and skill gaps through cocreation. While health systems increasingly rely on digital platforms to facilitate patient engagement, results demonstrate that usability challenges and complex design, often resulting from a failure to cocreate with users, are common friction points that inhibit successful digital health tool use. These barriers are not merely technical inconveniences; they are structural barriers that prevent users from accessing care and support. Prior studies have similarly identified usability challenges—such as manually entering data, following procedures to retrieve data (such as appointment details), and understanding medical terminology—as key barriers that prevent equitable use of patient portals among diverse populations [49,50].

To address usability challenges, vendors must adopt HCD principles and co-design strategies that engage diverse users throughout the design process to ensure that digital interfaces are intuitive, accessible, and responsive to users’ varying levels of digital proficiency. This includes incorporating plain language, reducing unnecessary steps, improving language functionalities for non-English speakers, and reducing privacy and security complexities that act as a barrier to users with low digital literacy [50]. Vendors must not assume a baseline level of digital literacy and should instead actively test for, design against, and consult patients about learnability, operability, and task complexity barriers in these digital health tools. By treating usability and accessibility as core priorities rather than downstream implementation concerns, vendors can play a pivotal role in promoting digital equity and closing gaps in health care access. For example, strict security needs for usernames and passwords (including activation codes and other 2-factor authentication) are not “user-friendly,” and while they make sense from a security perspective, they are advanced skills that many users do not understand or have experience with. Issues with complex password creation, entering an activation code, and other 2-factor authentication methods are demonstrated tension points for users from marginalized backgrounds who may have little experience in engaging with strict and complex privacy protocols. Vendors must include patient voices when weighing privacy concerns against usability to ensure that access and usability decisions do not further inequities [28,50].

In the meantime, without more usable products, health care systems need to be prepared to support patients in accessing and using existing digital health tools. As prior studies have shown, explicit identification of a “digital navigator” to support patients in using digital health tools facilitates equitable access and use of digital health tools [51,52]. Unfortunately, this technical assistance often falls to clinical care team members who are not trained to provide this type of support; as a result, patients do not receive adequate support, particularly in safety net systems [52,53]. Increasing support for digital navigator training and incorporating them as members of the clinical care team will be crucial to help overcome gaps in foundational skills and usability barriers.

### Limitations

We note several limitations of our study. We recruited participants from a large urban safety net health system that serves a diverse population, but our stratified samples based on core sociodemographic characteristics are small, limiting our ability to evaluate disparities in fundamental skills by sociodemographic characteristics. Additionally, the Hawthorne effect may have resulted in participants changing their behavior while completing the digital tasks because they were being observed. While the chosen tasks are essential in accessing and using digital health technology, they may not be representative of specific usability barriers that individuals face within the patient portal app itself (such as scheduling an appointment) or may be considered by some as antiquated processes (such as using a URL to access the patient portal). We used a patient portal emulator for this study, which was a web browser–based process; this may not reflect the same usability barriers that would exist for the use of a smartphone app. However, the fundamental skill challenges that were present for many patients would impact the use of a mobile app. Moreover, despite advances in digital health tool accessibility (translation to multiple languages and apps available on most devices), there are still limitations to the ability of digital health tools to be inclusive, resulting in issues that are not strictly due to lack of skills but are also influenced by language barriers or other sociodemographic barriers. However, our results will still provide further direction for future digital inclusion studies.

### Implications for Future Research

Future studies should more deeply examine usability issues of digital health tools, evaluating the tools themselves and not just tasks that are related to these tools. Future studies should also explore longitudinal improvements in digital skills following structured training interventions. Evidence has shown that provision of multimodal and audiovisual digital training is supported by patients and has been associated with improved use [45,46]. This evidence suggests that training among populations with marginalized identities can be beneficial, yet evidence is needed to assess training implications on digital skills. Additionally, assessing the impact of different instructional approaches—such as interactive tutorials versus guided assistance—could yield insights into optimizing digital literacy education.

Furthermore, there are benefits of improving digital literacy broadly, not only specific to digital health tools and digital health engagement. This study demonstrated that there are disparities in foundational skills that are not specific to digital health tools; therefore, designing collaborations with libraries or continuing education classes could serve as implementation strategies that can help mitigate the effects of these skill disparities and reduce digital exclusion.

### Conclusion

By integrating the results of the fundamental skills necessary to use digital health tools and usability barriers to using these tools, this study highlights critical gaps in digital literacy, particularly in device navigation and progressing through complex tasks. This study underscores the importance of usability-driven improvements to reduce digital barriers. By addressing these challenges through targeted interventions, digital accessibility may be enhanced, empowering users to navigate digital environments more effectively. Future work should explore user-tailored interventions aimed at providing individuals with the core fundamental skills necessary to use digital health tools, iterative usability testing to improve the usability of these tools, and personalized support mechanisms to support individuals as they navigate these tools. By systematically identifying and addressing these usability challenges, researchers and developers can create more inclusive digital health solutions that promote equitable health care access and improve patient outcomes across all populations.

## Supplementary material

10.2196/78430 Multimedia Appendix 1 Survey administered to participants.

10.2196/78430 Multimedia Appendix 2 Description of digital tasks and the coding matrix.

10.2196/78430 Multimedia Appendix 3 Qualitative codebook.

## Abbreviations

HCD: human-centered design
HCI: human-computer interaction
REDCap: Research Electronic Data Capture
SFHN: San Francisco Health Network
SMART Digital Health Scale: Skills Measurement and Readiness Training for Digital Health Scale
