# Supplementary material for: Identifying Skill and Usability Barriers to Digital Health Tool Use Among Older Adult Patients in US Safety Net Clinics: Mixed Methods Study
Source: JMIR Hum Factors. 2026 May 4;13:e78430. doi: 10.2196/78430 (PMC13138792; doi:10.2196/78430)
Supplement: Multimedia Appendix 1 [file humanfactors-v13-e78430-s001.docx]

## Multimedia Appendix 1

**APPENDIX 1. Selected Participant Questionnaire Items**

| **Question** | **Source** |  |
| --- | --- | --- |
| 1. What is your birthdate? [MM/DD/YYYY] 2. [If don’t know]: About how old are you? [age in years] | Self-developed |  |
| 1. What terms best express how you describe your gender identity?    1. Man    2. Woman    3. Non-binary    4. Transgender    5. Other, please specify _______________________    6. Prefer not to answer | Self-developed |  |
| 1. Are you of Hispanic, Latino, or Spanish origin?    1. No, not of Hispanic, Latino, or Spanish origin    2. Yes, Mexican, Mexican American, Chicano    3. Yes, Puerto Rican    4. Yes, Cuban    5. Yes, another Hispanic, Latino, or Spanish origin – Please specify, Salvadoran, Dominican, Colombian, Guatemalan, Spaniard, Ecuadorian, etc. | Self-developed |  |
| 1. What is your race? Mark one or more boxes AND print origins. 2. White, please specify: *(for example, German, Irish, English, Italian, Lebanese, Egyptian, etc.)* 3. Black or African American, please specify: *(for example, African American, Jamaican, Haitian, Nigerian, Ethiopian, Somali, etc.)* 4. American Indian or Alaska Native, please specify: *(for example, Navajo Nation, Blackfeet Tribe, Mayan, Aztec, Native Village of Barrow Inupiat Traditional Government, Nome Eskimo Community, etc.)* 5. Chinese 6. Filipino 7. Asian Indian 8. Vietnamese 9. Korean 10. Japanese 11. Other Asian, please specify: *(for example, Pakistani, Cambodian, Hmong, etc.)* 12. Native Hawaiian 13. Samoan 14. Chamorro 15. Other Pacific Islander, please specify: *(for example, Tongan, Fijian, Marshallese, etc.)* 16. Some other race, please specify: | Self-developed |  |
| 1. What is the highest grade or level of school you have completed or the highest degree you have received? 2. Never Attended/Kindergarten Only; 3. 1st Grade; 4. 2nd Grade; 5. 3rd Grade; 6. 4th Grade; 7. 5th Grade; 8. 6th Grade; 9. 7th Grade; 10. 8th Grade; 11. 9th Grade; 12. 10th Grade; 13. 11th Grade; 14. 12th Grade, No Diploma; 15. High School Graduate; 16. GED Or Equivalent; 17. Some College, No Degree; 18. Associate Degree: Occupational, Technical, Or Vocational Program; 19. Associate Degree: Academic Program; 20. Bachelor's Degree (Example: BA, AB, BS, BBA); 21. Master's Degree (Example: MA, MS, MEng, MEd, MBA); 22. Professional School Degree (Example: MD, DDS, DVM, JD); 23. Doctoral Degree (Example: PhD, EdD); 24. Refused; 25. DON'T KNOW | Self-developed |  |
| 1. Do you own a phone, tablet, or computer that can do a video call? 2. Yes 3. No 4. Don’t know/Not sure 5. Decline to state | Developed by Epic Workgroup focused on Digital Exclusion Screening Questions |  |
| 1. Do you have either Wi-Fi at home or an unlimited data plan (on your phone)? 2. Yes, Wi-Fi 3. Yes, unlimited data for phone 4. Yes, both Wi-Fi and unlimited data for phone 5. No 6. Don't know / Not sure 7. Decline to state | Internally developed at the San Francisco Health Network |  |
| 1. Are you enrolled in a patient portal (like MyChart) with your primary care provider?   [BARRIERS: PATIENT PORTAL]   1. Yes, at SFGH 2. Yes, at another health system 3. No 4. Don’t know/Not sure 5. Decline to state | | Self-developed |
