# Supplementary material for: Identifying Skill and Usability Barriers to Digital Health Tool Use Among Older Adult Patients in US Safety Net Clinics: Mixed Methods Study
Source: JMIR Hum Factors. 2026 May 4;13:e78430. doi: 10.2196/78430 (PMC13138792; doi:10.2196/78430)
Supplement: Multimedia Appendix 2 [file humanfactors-v13-e78430-s002.docx]

## Multimedia Appendix 2

**APPENDIX 2a. Detailed Descriptions of Digital Tasks.**

Each of the tasks were designed by the study team to imitate the actions necessary when joining a telemedicine call from a text message, searching for the patient portal by typing in the URL, creating a unique password for the patient portal, and logging in to the patient portal.

In the *first task (launching a video visit),* the participant received a standardized text message in English from an automated number containing an invitation to join the video visit by clicking the link in the text. Once the participant clicked the link, they followed a series of prompts to successfully join the video visit (e.g., allowing the camera and microphone access).

In the *second task (going to a specific health website),* the participant opened a web browser and typed in the URL for the patient portal website emulator into the URL/address bar.

In the *third task (signing in to a patient portal account via emulator),* the participant entered a provided username and password into the patient portal login fields to mimic logging in to the patient portal.

In the *fourth task (signing up for a patient portal account via emulator)*, participants created a patient portal account by entering an activation code, zip code, and birthdate, and then entering and provided username and creating a unique password for the account.

**APPENDIX 2b. Coding Matrix.**

Each column represents the possible codes that could be observed for each task, and rows represent individual participants. Observed codes for each participant during each task were documented in the appropriate cell, creating a matrix of observed codes across all tasks.

| **CODING MATRIX** | | | | | |
| --- | --- | --- | --- | --- | --- |
|  | **TASK 1: SIGN IN TO A VIDEO VISIT** | **TASK 2: GO TO A SPECIFIC WEBSITE (EMULATES MYCHART)** | **TASK 3: SIGN-IN TO MYCHART** | **TASK 4: MYCHART SIGN-UP** | |
|  | **A. Digital Skills** | **A. Digital Skills** | **A. Digital Skills** | **1. Follow steps in prompt** | **2. Review password creation** |
| Summary template: | *Device: what device is individual using*   *Challenges to look for: hesitation before starting task, hesitation between steps, hesitation with pop-up/text, hesitation with typing/password, rereading instructions, verbalizing instructions, typing errors, repeated errors, hunt and peck typing, lack of familiarity with keyboard characters, lack of familiarity with capitalization, verbalizing typing, lack of familiarity with phone layout, lack of familiarity with apps, extraneous scrolling, extraneous tapping, ignoring pop-ups/prompts, choosing wrong link/menu, asking for assistance, not understanding the objective of the task, not understanding layout of information*   *Success to look for: continuous progress of task, lack of hesitation, no errors, error and self-correction, advanced typing skills, advanced navgiation skills, advanced phone setting skills, minimal scrolling* | *Device: what device is individual using*   *Challenges to look for: hesitation before starting task, hesitation between steps, hesitation with pop-up/text, hesitation with typing/password, rereading instructions, verbalizing instructions, typing errors, repeated errors, hunt and peck typing, lack of familiarity with keyboard characters, lack of familiarity with capitalization, verbalizing typing, lack of familiarity with phone layout, lack of familiarity with apps, extraneous scrolling, extraneous tapping, ignoring pop-ups/prompts, choosing wrong link/menu, asking for assistance, not understanding the objective of the task, not understanding layout of information*   *Success to look for: continuous progress of task, lack of hesitation, no errors, error and self-correction, advanced typing skills, advanced navgiation skills, advanced phone setting skills, minimal scrolling* | *Device: what device is individual using*   *Challenges to look for: hesitation before starting task, hesitation between steps, hesitation with pop-up/text, hesitation with typing/password, rereading instructions, verbalizing instructions, typing errors, repeated errors, hunt and peck typing, lack of familiarity with keyboard characters, lack of familiarity with capitalization, verbalizing typing, lack of familiarity with phone layout, lack of familiarity with apps, extraneous scrolling, extraneous tapping, ignoring pop-ups/prompts, choosing wrong link/menu, asking for assistance, not understanding the objective of the task, not understanding layout of information, dummy account issues, lack of familiarity with password setting, use incorrect password, use incorrect username*   *Success to look for: continuous progress of task, lack of hesitation, no errors, error and self-correction, advanced typing skills, advanced navgiation skills, advanced phone setting skills, minimal scrolling, advanced username/pw skills* | *Device: what device is individual using*   *Challenges to look for: hesitation before starting task, hesitation between steps, hesitation with pop-up/text, hesitation with typing/password, rereading instructions, verbalizing instructions, typing errors, repeated errors, hunt and peck typing, lack of familiarity with keyboard characters, lack of familiarity with capitalization, verbalizing typing, lack of familiarity with phone layout, lack of familiarity with apps, extraneous scrolling, extraneous tapping, ignoring pop-ups/prompts, choosing wrong link/menu, asking for assistance, not understanding the objective of the task, not understanding layout of information*   *Success to look for: continuous progress of task, lack of hesitation, no errors, error and self-correction, advanced typing skills, advanced navgiation skills, advanced phone setting skills, minimal scrolling* | *Device: what device is individual using*   *Challenges to look for: hesitation before starting task, hesitation between steps, hesitation with pop-up/text, hesitation with typing/password, rereading instructions, verbalizing instructions, typing errors, repeated errors, hunt and peck typing, lack of familiarity with keyboard characters, lack of familiarity with capitalization, verbalizing typing, lack of familiarity with phone layout, lack of familiarity with apps, extraneous scrolling, extraneous tapping, ignoring pop-ups/prompts, choosing wrong link/menu, asking for assistance, not understanding the objective of the task, not understanding layout of information, dummy account issues, lack of familiarity with password setting, use incorrect password, use incorrect username*   *Success to look for: continuous progress of task, lack of hesitation, no errors, error and self-correction, advanced typing skills, advanced navgiation skills, advanced phone setting skills, minimal scrolling, advanced username/pw skills* |
| Participant 1 | Coder 1:  Coder 2: | Coder 1:  Coder 2: | Coder 1:  Coder 2: | Coder 1:  Coder 2: | Coder 1:  Coder 2: |
