# Supplementary material for: Identifying Skill and Usability Barriers to Digital Health Tool Use Among Older Adult Patients in US Safety Net Clinics: Mixed Methods Study
Source: JMIR Hum Factors. 2026 May 4;13:e78430. doi: 10.2196/78430 (PMC13138792; doi:10.2196/78430)
Supplement: Multimedia Appendix 3 [file humanfactors-v13-e78430-s003.docx]

## Multimedia Appendix 3

**APPENDIX 3. Qualitative Codebook**

| **Grouping** | **Code** | **Definition** |
| --- | --- | --- |
| Novice Keyboard Skills | Unfamiliarity with capitalization | Individual demonstrates lack of familiarity with capitalization, either by vocalizing it or by not knowing how to do it |
|  | Unfamiliarity with special keys | Individual demonstrates lack of familiarity with keyboard characters, either by vocalizing it or by using the wrong characters |
| Inefficient Typing | Hunt and peck typing | Individual types with one finger (or with a stylus) at a time, “hunting” for each letter on the keyboard; can also verbalize searching for letters |
| Advanced typing skills | Advanced typing skills | Individual displays advanced typing skills (intuitive navigation of the keyboard, understanding of capitalization & special keys) |
| Inefficient Navigation | Extraneous scrolling | Individual scrolls excessively, often too much and past the field or objective they are looking for (also if they scroll incorrectly, such as trying to use the laptop screen as a touchscreen and trying to scroll that way) |
|  | Extraneous tapping | Individual taps their device excessively and maybe arbitrarily trying to click on different things or unsure how to tap (also if they use laptop as a touchscreen and try to tap on monitor) |
| Novice Device Skills | Unfamiliarity with phone layout (more general) | Individual expresses or demonstrates lack of familiarity with the phone layout, doesn’t understand where to go or what to do, how to navigate different steps |
|  | Unfamiliarity with apps (location/what they look like) | Individual expresses or demonstrates lack of familiarity with device applications, such as where they are or how to find them, what they look like, when to use which app, etc. |
|  | Unfamiliarity with trackpad | Individual expresses or demonstrates lack of familiarity with using a trackpad or touchscreen, such as controlling a cursor or how to select items (or using it appropriately, i.e., if a user mistakenly treats the screen of a laptop as a touchscreen) |
| Advanced Navigation & Device Skills | Minimal scrolling | Individual demonstrates minimal (or lack of excessive) scrolling when completing task |
|  | Advanced phone navigation skills | Individual displays advanced and intuitive navigation skills and understands how to navigate different apps or steps |
|  | Advanced phone setting skills | Individual displays skills adjusting or changing any settings that might be needed |
| Continuous Progression of Tasks | Continuous progress of task | Individual progresses through task without any breaks in performance flow |
| Unsure how to proceed | Unsure how to proceed | Individual hesitates between steps/before moving to the next step (i.e., hesitates before pushing enter, double checks their work, etc.) |
| Asking for Assistance | Asking for assistance | Individual requests assistance with the task, whether or not assistance is provided |
| Ineffective interaction with Pop-ups/prompts | Choosing wrong link/pop-up/menu | Individual chooses the wrong link, pop-up, or menu during the task |
|  | Ignoring pop-ups/prompts | Individual ignores pop-ups or prompts (or closes them) when they are relevant to completing the task |
| Finding/Using web browser | Finding/Using web browser | Individual struggles with navigating a web browser, either verbalizes confusion when prompted to use it, expresses hesitation with using a web browser, or navigates in effectively |
| Novice URL skills | Novice URL skills | Individual does not understand how to type in a URL, either verbally expresses confusion, types it into the wrong place, incorrectly uses autocorrect to finish the URL, or other ineffective URL strategy |
| Novice password skills | Novice password skills | Individual demonstrates or expresses lack of familiarity with password setting (such as asking questions about it, not sure what to put or how to make a password, etc.) |
| Learnability | Confusion/Hesitation with text message | Individual hesitates or is confused with the text message, selecting the link, responding to it, ignoring it, etc.; either in action or verbally expresses it |
|  | Mistake search bar for URL bar | Individual types the URL into the search bar and does not know what to do after |
|  | Not knowing which link to select | Individual hesitates with a pop-up or selecting a link, unsure which one to choose or selecting the wrong one |
|  | How to do manual credential entry | Individual uses incorrect username/password/activation code (i.e., their password instead of the dummy password, doesn’t type in the full password, etc.) |
| Operability | Repeated errors | Individual makes repeated errors due to non-specific error or login failed notifications  NOTE: specify where the repeated errors occurred and whether notification said “error” or “login failed” |
